# Brain network and energy imbalance in Parkinson’s disease: linking ATP reduction and *α*-synuclein pathology

**DOI:** 10.3389/fnmol.2024.1507033

**Published:** 2025-01-22

**Authors:** Hirohisa Watanabe, Sayuri Shima, Kazuya Kawabata, Yasuaki Mizutani, Akihiro Ueda, Mizuki Ito

**Affiliations:** ^1^Department of Neurology, School of Medicine, Fujita Health University, Toyoake, Japan; ^2^Department of Neurology, Fujita Health University Okazaki Medical Center, Okazaki, Japan; ^3^Department of Neurology, Fujita Health University Bantane Hospital, Nagoya, Japan

**Keywords:** ATP metabolism, cortical hubs, mitochondrial dysfunction, *α*-synuclein aggregation, energy imbalance, hypoxanthine

## Abstract

Parkinson’s disease (PD) involves the disruption of brain energy homeostasis. This encompasses broad-impact factors such as mitochondrial dysfunction, impaired glycolysis, and other metabolic disturbances, like disruptions in the pentose phosphate pathway and purine metabolism. Cortical hubs, which are highly connected regions essential for coordinating multiple brain functions, require significant energy due to their dense synaptic activity and long-range connections. Deficits in ATP production in PD can severely impair these hubs. The energy imbalance also affects subcortical regions, including the massive axonal arbors in the striatum of substantia nigra pars compacta neurons, due to their high metabolic demand. This ATP decline may result in *α*-synuclein accumulation, autophagy-lysosomal system impairment, neuronal network breakdown and accelerated neurodegeneration. We propose an “ATP Supply–Demand Mismatch Model” to help explain the pathogenesis of PD. This model emphasizes how ATP deficits drive pathological protein aggregation, impaired autophagy, and the degeneration of key brain networks, contributing to both motor and non-motor symptoms.

## Introduction

The identification and understanding of various brain functions have significantly evolved through techniques correlating symptoms with lesions in specific brain areas (Fox, 2018). However, identical symptoms can result from entirely different lesions, and multiple higher brain functions can be impaired by the same cortical or subcortical lesions (Corbetta et al., 2015). The concept of the connectome aims to comprehensively understand brain function from a network perspective by mapping the entire neuronal network (Sporns et al., 2005). This approach has rapidly gained popularity, driven by advancements in computer processing power (Greicius et al., 2003).

The human brain is often conceptualized as a communication network composed of neurons and neural circuits. Within this network, certain regions act as hubs, where interactions between neurons and different neural circuits are especially abundant. These hub regions are characterized by their high connectivity to other areas of the brain, playing a crucial role in coordinating multiple functions. The brain network exhibits both scale-free and small-world properties: it has a few highly connected hubs (scale-free) and maintains short path lengths with high clustering, which facilitates efficient information transfer (small-world) (Stam, 2014). The importance of these hubs becomes evident when considering the dramatic reduction in the network’s overall efficiency if they are impaired (Crossley et al., 2014). Moreover, hubs are central to the brain’s compensatory mechanisms during normal aging and are frequent sites of age-related neurodegenerative disorders (Watanabe et al., 2021).

Approximately 64% of the brain’s energy is consumed by synaptic activity, and hub regions—rich in synapses—are therefore thought to have especially high energy demands (Sengupta et al., 2013). They also contain many long axons that require large amounts of energy. In mice, genes related to ATP synthesis and metabolic regulation show particularly high expression in these regions, suggesting enhanced metabolic activity that supports the high energy demands of these hub areas (Fulcher and Fornito, 2016). Similarly, glucose metabolism is particularly active in the hub regions of the human brain (Tomasi et al., 2013). These findings highlight that hub regions are key sites of high energy demand in the brain.

Mitochondrial ATP production rate per gram of skeletal muscle in adult humans was estimated to decline by approximately 8% every 10 years (Short et al., 2005). Also in the brain, mitochondrial function, including ATP production and oxidative phosphorylation, declines significantly with age due to accumulated oxidative damage and mitochondrial DNA mutations, leading to energy deficits (Bartman et al., 2024). This persistent energy gap—caused by an imbalance between energy demand and supply, along with neuropathological processes—creates a vicious cycle of energy depletion and further dysfunction, contributing to cognitive decline and neurodegenerative diseases (Cunnane et al., 2020). Therefore, in advanced age, the hub regions are constantly on the brink of an energy crisis.

Parkinson’s disease (PD) is the second most common neurodegenerative disease after Alzheimer’s disease. Patients with PD show disruption of the cortical hub regions, as assessed by resting state functional MRI studies (Koshimori et al., 2016; Kawabata et al., 2018, 2020; Wolters et al., 2019; Bagarinao et al., 2022). Cortical hub disruptions in PD primarily consist of decreased functional connectivity between these hubs and other brain regions, which reduces the efficiency of neural communication. Cortical hubs, essential for integrating and distributing information across neural networks, lose their integrative capacity, resulting in fragmented network activity (Stam, 2014). Furthermore, these hubs exhibit abnormal neural activity patterns, including reduced synchronization and altered metabolic activity, which further compromise their function (Tomasi et al., 2013). Such disruptions impair both global and local information processing, directly contributing to the motor and cognitive symptoms characteristic of PD. These include tremors, rigidity, slowed information processing, and executive dysfunction (Bagarinao et al., 2022; Wolters et al., 2019). More recently, we proposed that the extensive branching of substantia nigra pars compacta (SNc) neurons in the striatum, similar to the cortical hub regions, increases vulnerability to imbalances between energy supply and demand, which may promote neurodegeneration in PD (Watanabe et al., 2024).

The mitochondrial tricarboxylic acid (TCA) cycle and glycolysis are critical for cellular energy production (Johnson et al., 2019; Cunnane et al., 2020). Abnormalities in mitochondrial function have been observed not only in sporadic cases but also in familial PD, and it has been reported that mitochondrial dysfunction can be observed even before cell death or the deposition of *α*SN aggregates (Toomey et al., 2022). Mitochondrial dysfunction is thought to be associated with the pathogenesis of PD through mechanisms such as selective autophagy of damaged mitochondria, impaired fusion and division of mitochondria, increased reactive oxygen species, oxidative stress, and iron-dependent cell death (Gao et al., 2022). In addition, mitochondrial damage is closely associated with impaired ATP production.

Recent evidence suggests that glycolysis plays a significant role in PD pathology, with impaired glycolytic flux contributing to reduced ATP production and neuronal dysfunction (Naeem et al., 2022; Mischley et al., 2023). Studies have shown that enhanced glycolysis can support mitochondrial function and protect dopaminergic neurons from degeneration (Cai et al., 2019). A recent study showed that phosphoglycerate kinase 1 (PGK1) is crucial for neuronal glycolysis and that increasing its expression enhances ATP production (Kokotos et al., 2024). Furthermore, enhancing PGK1 activity has been shown to increase brain ATP levels and slow or prevent neurodegeneration in PD models by stimulating glycolysis and supporting mitochondrial function (Cai et al., 2019). A meta-analysis also found that glycolysis-enhancing drugs, such as PGK1 activators, were associated with a lower incidence of PD, further supporting the neuroprotective potential of these pathways (Ribeiro et al., 2024). Table 1 summarizes an overview of the ATP metabolism pathway in the brain and their disruption in PD. These findings suggest that targeting bioenergetic deficits may be a promising approach for PD treatment.

**Table 1:** Overview of ATP metabolism pathways in the brain and their disruption in PD.

Pathway	Key enzymes/components	Main products	Role in energy production	Disruption in PD
Mitochondrial function	Electron transport chain (RTC) complexes, ATP synthase, pyruvate dehydrogenase	ATP, reactive oxygen species (ROS)	Main source of ATP via oxidative phosphorylation; mitophagy (removal of damaged mitochondria) is essential for maintaining mitochondrial health and cellular energy balance	Mitochondrial dysfunction due to impaired mitophagy, leading to reduced ATP production, increased ROS, oxidative stress, and accumulation of damaged mitochondria
Glycolysis	Hexokinase, phosphofructokinase, phosphoglycerate kinase (PGK1), lactate dehydrogenase	Pyruvate, ATP, NADH	Provides ATP for rapid energy production; supports synaptic vesicle recycling, lactate production for neuron support, and supplies metabolic intermediates for other pathways	Impaired glycolytic flux due to enzyme dysfunction, reduced ATP synthesis; affects synaptic function, lactate support, and disrupts downstream metabolic pathways in PD
Pentose phosphate pathway	Glucose-6-phosphate dehydrogenase (G6PD), transketolase	NADPH, ribose-5-phosphate	Generates NADPH for antioxidant defense, nucleotide synthesis, and cellular redox balance	Altered G6PD activity in PD affects NADPH production, leading to oxidative stress and impaired neuronal survival
ATP recycling	Adenylate kinase, AMP deaminase, hypoxanthine-guanine phosphoribosyltranferase (HGPRT)	ATP, IMP, hypoxanthine, inosine	Resynthesizes ATP from ADP and AMP; hypoxanthine is a key intermediate for ATP generation in neurons through the purine salvage pathway	Decrease hypoxanthine levels impair ATP recycling, resulting, in energy deficits and promoting *α*-synuclein aggregation in pD

PD, Parkinson’s disease; ATP, adenosine triphosphate; ADP, adenosine diphosphate; AMP, adenosine monophosphate; IMP, Inosine monophosphate; NADH, Nicotinamide adenine dinucleotide; NADPH, Nicotinamide adenine dinucleotide phosphate.

However, in PD, the answers to fundamental questions such as (1) what kind of clinical picture or imaging findings are associated with impaired brain energetics, (2) whether there are disorders in energy production systems other than mitochondria and glycolysis, such as the purine metabolite pathway, and (3) how impaired brain energetics cause pathological protein accumulation have not been found. In this review, we summarize the latest findings on these questions in the context of PD and consider the potential for developing new treatments based on these findings and the potential for application to other neurodegenerative disorders associated with aging.

### *α*-synuclein: role, diagnosis, and unresolved questions in PD

The pathological loss of nigrostriatal neurons is a characteristic finding in patients with PD associated with motor symptoms such as resting tremor, bradykinesia, and rigidity (Samii et al., 2004). It has been shown that bradykinesia (Bologna et al., 2020) and resting tremor (Helmich et al., 2012) involve the sensorimotor cortex, cerebellum, and thalamus from the early stages of the disease. As the disease progresses, lesions spread to the cerebral cortex, causing hallucinations and cognitive decline (Aarsland et al., 2017). Pathologically, it has been reported that changes in neuronal neurofilament light chains and myelin proteins occur in the motor cortex even before the appearance of motor symptoms (Fu et al., 2022). These findings suggest that a wide range of brain regions beyond the substantia nigra and striatum are already involved during an early PD stage from the network perspective.

*α*-synuclein (*α*SN) is the main component protein of Lewy bodies, which are classically important in diagnosing PD. Recently, it has become possible to assess the presence of misfolded *α*SN in the cerebrospinal fluid and blood using the *α*SN seed amplification assay (SAA), and biological diagnostic criteria that combine the presence of misfolded *α*SN, neurodegeneration, and genetic profiles have been proposed (Höglinger et al., 2024). A cross-sectional study using the *α*SN SAA showed high sensitivity and specificity in differentiating PD from healthy individuals (Siderowf et al., 2023). However, results obtained from *α*-SAA have not been proven to reflect the origin of neurodegeneration ultimately or be a marker for its progression (Obeso and Calabressi, 2024).

The fundamental question of how *α*SN aggregation causes neurodegeneration remains unresolved. The timing of *α*SN aggregation and PD cell death is unknown, and the toxicity of *α*SN has only been proven in animal models. There are familial forms of PD in which *α*SN accumulation is not required for neurodegeneration, and the correlation between *α*SN accumulation and neurodegeneration is unknown (Lang and Espay, 2018). In addition, a study comparing diffusion tensor MRI and *α*SN deposition in postmortem brains found that the correlation between *α*SN deposition and local changes in brain networks is limited (Frigerio et al., 2024).

Furthermore, antibody therapy for *α*SN such as prasinezumab and cinpanemab has not shown any therapeutic effects (Lang et al., 2022; Pagano et al., 2022). Both are monoclonal antibodies targeting *α*SN, a critical protein in PD pathology, but their mechanisms of action differ. Prasinezumab targets oligomerized and aggregated forms of *α*SN to reduce the propagation of pathological aggregates between neurons, focusing on extracellular aggregates to prevent their toxic effects and spread. On the other hand, cinpanemab targets the soluble monomeric form of *α*SN to prevent the earliest stages of aggregation, aiming to inhibit the initial misfolding events. These distinctions highlight two complementary therapeutic strategies to address different phases of *α*SN pathology in PD. Although it has been suggested that prasinezumab may be effective against the diffuse malignant form of PD—which refers to an aggressive and rapidly progressing subtype of the disease characterized by widespread neurodegeneration (Pagano et al., 2024)—there were no significant changes in clinical indicators, dopamine transporter SPECT, MRI, neurofilament light chain, or other PD biomarkers following administration of cimpanema (Hutchison et al., 2024). The idea has also been proposed that the loss of functional *α*SN may affect pathogenesis (Espay and Lees, 2024).

In summary, despite advances in understanding the role of *α*SN in PD, its exact contribution to neurodegeneration remains unclear. In particular, the process by which physiological *α*SN becomes toxic remains unknown. The limitations of current biomarkers and the lack of efficacy in *α*SN-targeted therapies highlight the need for further research into their complex pathology and potential impact on disease progression.

### Beyond *α*SN: association of impaired brain energetics with clinical, imaging, and neurophysiological findings in Parkinson’s disease

Impaired brain energetics can relate to the motor and nonmotor symptoms of PD. For instance, parkinsonism occurs in 6–13% of individuals with primary mitochondrial disease, and they are referred to as having mitochondrial parkinsonism (Lopriore et al., 2024). Patients with PD commonly show weight loss and low body mass index related to loss of fat mass (Lorefält et al., 2009; Yong et al., 2020). In PD, motor symptoms often worsen during systemic infections, which may be caused by alterations in mitochondrial metabolism and increased oxidative stress triggered by the infection (Brugger et al., 2015).

Neuroimaging obtained with [18F]-fluorodeoxyglucose (FDG) positron emission tomography (PET)—a technique that monitors glucose metabolism in the brain as an indicator of neuronal activity—and analyzed using a scaled subprofile model and principal component analysis (SSM PCA), revealed a PD-related pattern (PDRP). This pattern was characterized by relatively increased metabolism in the thalamus, globus pallidus/putamen, cerebellum, and pons, along with relative hypometabolism in the occipital, temporal, parietal, and frontal cortices as compared with healthy control individuals (Meles et al., 2020). The PDRP scores were significantly correlated with motor symptoms and increased with disease progression.

A network metric called “functional connectivity overlap ratio analysis,” which uses resting-state functional MRI data to assess how different brain regions communicate with each other (Bagarinao et al., 2020), showed significant PD-associated alterations in connector hubs identified in the sensorimotor cortex and cerebellum (Bagarinao et al., 2022). These alterations correlate with motor symptoms such as tremor, postural instability, and gait disturbances in PD.

Gamma oscillations (30–100 Hz) synchronize excitatory neurons and networks, relying on fast-spiking GABAergic interneurons with high mitochondrial density and specialized myelination. These interneurons have high metabolic needs, are primarily supported by oxidative phosphorylation, and are particularly sensitive to energy and oxygen deficits (Kann, 2016; Oyarzabal and Marin-Valencia, 2019). A transcranial alternating current stimulation study showed that abnormal *β* and *γ* oscillations at the primary motor cortex level of the basal ganglia-thalamo-cortical network play a relevant role in the pathophysiology of bradykinesia in PD (Guerra et al., 2022).

Magnetic resonance spectroscopy (MRS) used to measure mitochondrial ATP production in hand and leg muscles revealed decreases in ATP production and NAD levels in the tibialis anterior, along with reductions in muscle endurance and specific force, suggesting that reduced ATP production contributes to muscle fatigue in PD (Mischley et al., 2023).

Levodopa (L-dopa) is the mainstay of PD therapy. L-dopa is absorbed in the gastrointestinal tract, crosses the blood–brain barrier, and is converted into dopamine by the enzyme aromatic L-amino acid decarboxylase. This supplementation of dopamine helps mitigate motor symptoms such as bradykinesia, rigidity, and tremor. The relationship between levodopa (L-dopa) administration and energy metabolism in PD is complex and multifaceted. L-dopa might exacerbate mitochondrial dysfunction, as demonstrated by studies showing a reduction in high-energy phosphorus-containing metabolites in the basal ganglia (Prasuhn et al., 2024). However, evidence also indicates that levodopa may improve PD-related patterns in [18F]-FDG PET studies (Asanuma et al., 2006), possibly by ameliorating pathological hyperactivity or abnormal metabolic patterns. It has been proposed that L-dopa may enhance several compensatory processes in response to dopamine deficiency (Blesa et al., 2017), and this may potentially offset energy deficits. In this context, we should consider that ATP in neurons is mainly used by Na^+^/K^+^-ATPase and Ca^2+^-ATPase, which are cell membrane pumps that readjust ion gradients during neuronal activity (Magistretti and Allaman, 2018; Bordone et al., 2019; Oyarzabal and Marin-Valencia, 2019).

Approximately 30–80% of patients with PD show dementia during their illness. The causes are heterogeneous, but atypical FDG patterns—such as DLB-like patterns, characterized by temporal–parietal and occipital hypometabolism, and variably associated with frontal hypometabolism at baseline—can predict the risk of dementia in PD (Pilotto et al., 2018). According to a recent review (Zarkali et al., 2024), resting-state functional MRI studies revealed an overall increase in local connectivity accompanied by a reduction in long-range functional connectivity (Baggio et al., 2015), a temporal shift toward more segregated functional connectivity (Díez-Cirarda et al., 2018; Fiorenzato et al., 2019) and decoupling between functional and underlying structural connectivity in transmodal (higher association) regions (Zarkali et al., 2021).

**Figure 1:**
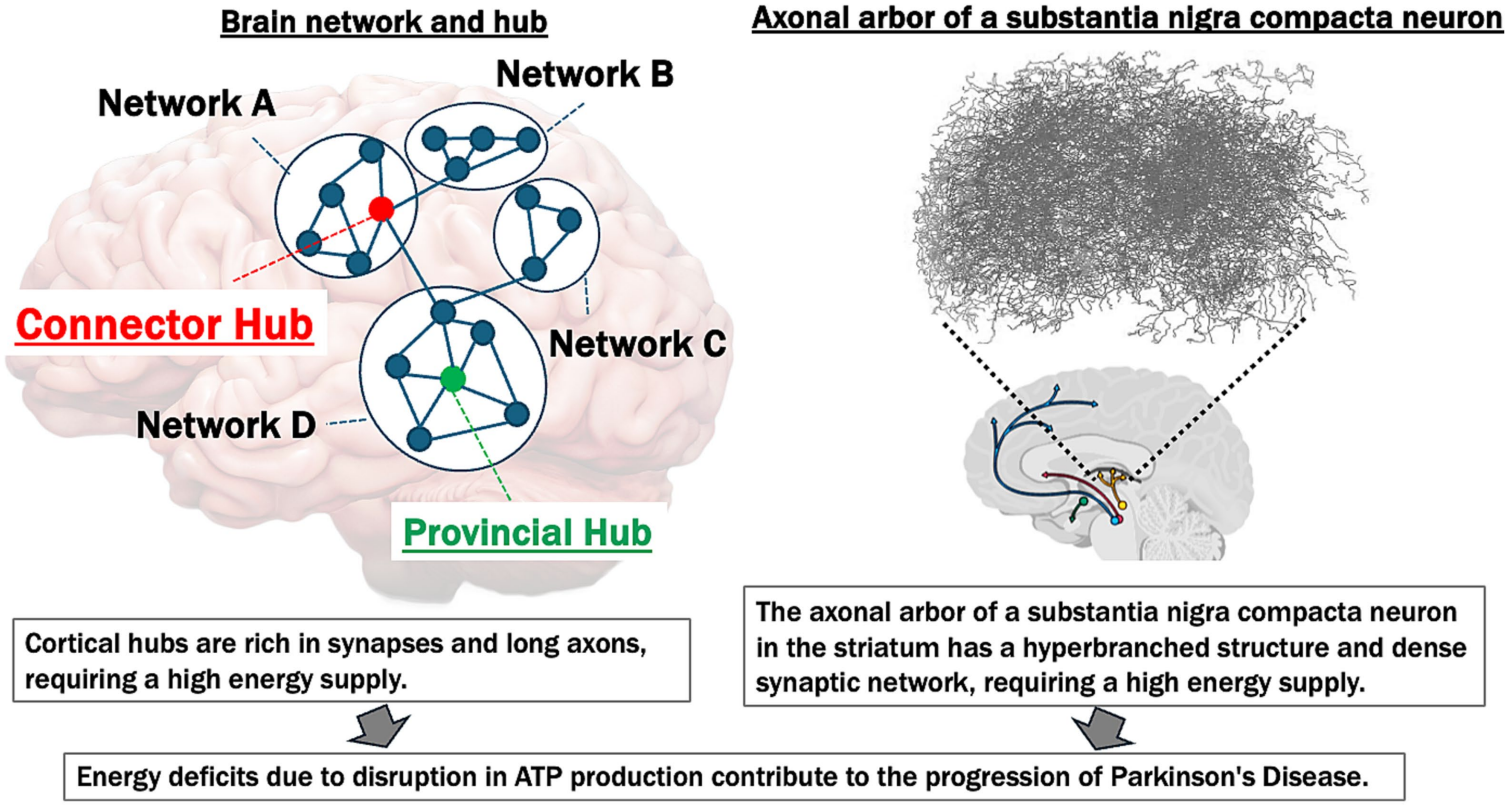
Common vulnerabilities to energy deficits of brain hubs and substantia Nigra in Parkinson’s disease. This figure illustrates the high energy demands of both cortical network hubs and the substantia Nigra, emphasizing their vulnerability to ATP deficits and their contribution to the progression of Parkinson’s disease (PD). Left panel: the brain network hubs (connector and provincial hubs) are rich in synapses and possess long axons, making them particularly energy-intensive. The “Connector Hub” (marked in red) integrates information across different networks (A, B, C, D), while the “Provincial Hub” (marked in green) connects nodes within a single network. These hubs require substantial ATP to support synaptic activity and maintain network integrity. Disruption in ATP production leads to energy deficits, contributing to the breakdown of network connectivity and the progression of neurodegenerative diseases. Right panel: the axonal arbor in the striatum of a typical substantia nigra dopaminergic neuron displays a hyperbranched structure with a dense synaptic network (figure modified from Matsuda et al., 2009), indicative of high metabolic and energy requirements. This region is particularly susceptible to energy supply deficiencies. In PD, ATP reduction and subsequent energy deficits exacerbate neuroinflammation, oxidative stress, and neural dysfunction, which accelerate disease progression.

In summary, FDG PET, MRI, MRS, and electrophysiological studies underscore the central role of impaired brain energetics and altered brain network connectivity in the pathogenesis and progression of PD. Disruptions in energy metabolism, functional connectivity, and compensatory mechanisms contribute to clinical symptoms—including motor dysfunction, cognitive decline, and variable responses to treatments such as L-dopa. Figure 1 illustrates common vulnerabilities to energy deficits of brain hubs and the substantia nigra in PD.

### Impaired brain energetics beyond mitochondria: pentose phosphate pathway (PPP) disruption in PD

The pentose phosphate pathway (PPP), located in the cytoplasm of cells, can be divided into two main phases: oxidative and nonoxidative. The oxidative phase involves the oxidation of glucose-6-phosphate (G6P) by the enzyme glucose-6-phosphate dehydrogenase (G6PD), producing NADPH and ribulose-5-phosphate. NADPH is crucial for maintaining the cell’s redox balance and combating oxidative stress. In the non-oxidative phase, ribulose-5-phosphate is converted to ribose-5-phosphate (R5P) by isomerase. R5P has three main roles in cellular metabolism: (i) it can be converted back to glucose-6-phosphate (G6P) via transketolase (TKT) to maintain the pentose phosphate pathway (PPP) and redox balance, (ii) it can be utilized in glycolysis to produce energy, or (iii) it can be used in purine nucleotide synthesis by forming inosine monophosphate (IMP) through phosphoribosyl pyrophosphate (PRPP). This flexibility helps cells to adapt their metabolic processes to meet demands for energy, antioxidants, and nucleotides.

The oxidative phase of PPP appears to be impaired in patients with PD. A study using 6-amino-nicotinamide adenine dinucleotide (6-ANAD) as an inhibitor of PPP demonstrated that decreased NADPH production led to reduced levels of biopterin. This reduction impairs tyrosine hydroxylase, the rate-limiting enzyme in dopamine synthesis, contributing to dopamine deficiency in rat model of PD (Herken, 1990).

Postmortem studies of brain tissue from patients with PD have shown decreased G6PD levels in the putamen during the early stages of PD and in the cerebellum during both early and late stages. These reductions are thought to be linked to impaired antioxidant responses. Interestingly, increased NADPH production was observed in affected brain regions of late-stage PD patients, suggesting a compensatory response to oxidative stress. G6PD levels were also elevated in the cerebral cortex of patients with moderate-to-severe PD (Dunn et al., 2014).

In contrast to the findings in human patients, several animal models of PD, including those based on intranigral and intraperitoneal injections of lipopolysaccharide (LPS), MPTP exposure, or A53T *α*-synuclein transgenics, showed increased G6PD expression and activity (Tu et al., 2019). This elevation correlates with microglial activation and the subsequent loss of dopaminergic neurons in the substantia nigra. Microglia with elevated G6PD activity produce excessive NADPH, fueling NADPH oxidase (NOX2)-mediated ROS production. Pharmacological inhibitors of G6PD, such as 6-aminonicotinamide and dehydroepiandrosterone, or siRNA-mediated G6PD knockdown, successfully reduced microglial activation and oxidative stress. These interventions protected dopaminergic neurons from degeneration in both *in vitro* and *in vivo* models of PD (Camandola and Mattson, 2017; Tu et al., 2019). Additionally, G6PD inhibition suppressed NF-κB pathway activation, indicating that G6PD influences oxidative stress and inflammatory responses (Tu et al., 2019). NADPH has a dual role; it can reduce oxidative stress and support antioxidant defenses, but it also plays a crucial role in inflammation through ROS generation and microglial activation (Ransohoff, 2016).

In addition to oxidative phase impairments, PD disrupts the non-oxidative phase of PPP. Ribose-5-phosphate (R5P), a product of PPP, serves as a precursor for purine nucleotide synthesis, producing inosine monophosphate (IMP) and its metabolites. Recent research has shown that inosine, a downstream metabolite of IMP, is significantly decreased in the cerebrospinal fluid (CSF) of patients with PD, indicating a potential disruption in purine metabolism (Shima et al., 2024). This finding points to an imbalance in the non-oxidative phase of PPP and its downstream metabolic pathways, further contributing to the metabolic dysregulation observed in PD.

Epidemiological studies have shown that paraquat is a significant environmental risk factor for PD, with evidence suggesting that exposure to this herbicide can lead to selective dopaminergic neuron degeneration (Dorsey and Ray, 2023). Paraquat exposure induces significant changes in glucose metabolism, increases glucose uptake, and redirects glucose into the PPP (Powers et al., 2017). This shift enhances the production of NADPH, which paraquat utilizes for its redox cycling, which generates reactive oxygen species (ROS) and causes oxidative stress. By “hijacking” the PPP, paraquat increases G6PD activity, a key enzyme in the pathway. This hijacking leads to the accumulation of PPP metabolites, elevated NADPH production, and an imbalance in the cellular redox status, ultimately contributing to cell death. Intriguingly, the olfactory bulb—one of the earliest and most commonly affected regions in PD—has been shown to exhibit significantly higher activity of G6PD and another enzyme, 6-phosphogluconate dehydrogenase, compared to the cortex, hippocampus, striatum, or septum, with levels in the olfactory bulb being four times higher in a rat study (Ninfali et al., 1997). However, the relationship between paraquat, PPP, G6PD, and *α*SN remains unclear. In this context, some studies have investigated the interaction between PQ and *α*SN aggregation, suggesting that their synergistic effects alter glucose metabolism and affect cellular energy production (Anandhan et al., 2017). Additionally, glucose metabolism or AMPK signaling inhibition has been reported to mitigate the combined toxic effects of PQ and *α*-SN aggregation (Anandhan et al., 2017).

### Impaired brain energetics other than mitochondria: impairments of ATP recycling

Adenosine triphosphate (ATP) is the “energy currency” of the cell. During intense energy demands, such as extreme exercise, cells enhance the adenylate kinase reaction (2 ADP→ATP + AMP) by utilizing AMP deaminase, which degrades AMP to IMP. IMP is further broken down into inosine, followed by the degradation of purines through the following pathway: hypoxanthine→xanthine→urate (Johnson et al., 2019). Over 90% of hypoxanthine is converted to IMP via hypoxanthine-guanine phosphoribosyltransferase (HPRT) and re-synthesized into ATP via IMP (Murray, 1971). Xanthine oxidoreductase inhibitors, which reduce the breakdown of hypoxanthine and xanthine, have been reported to reduce PD incidence (Song et al., 2023). We recently found that hypoxanthine levels in the serum and cerebrospinal fluid and inosine levels in cerebrospinal fluid significantly decreased in idiopathic PD (Shima et al., 2024).

As inosine is a metabolite of IMP, it may reflect decreased purine levels in the brain. Reduced serum hypoxanthine levels in patients with PD have been reported in patients with LRRK2 mutations (Johansen et al., 2009). Serum hypoxanthine is primarily produced in muscles (The Parkinson Study Group SURE-PD Investigators et al., 2014) and adipose tissue (Furuhashi, 2020). Therefore, reduced physical activity (van Nimwegen et al., 2011) and lipid system abnormalities (Galper et al., 2022) observed in patients with PD may contribute to lower hypoxanthine levels. While ATP levels in brain cells cannot be measured directly, we assume that a decrease in hypoxanthine in the serum and cerebrospinal fluid may correspond with a reduction in brain ATP.

HPRT deficiency, known to cause Lesch–Nyhan syndrome, has been shown in a study using induced pluripotent stem cells (iPSCs) to lead to ATP depletion in dopaminergic progenitor cells, which in turn impairs neuronal maturation (Bell et al., 2021). Since HPRT1 plays a critical role in the purine salvage pathway, its deficiency results in a significant reduction in purine derivatives, including ATP. Metabolic analysis revealed that glucose is increasingly diverted into the PPP to support *de novo* purine synthesis, further decreasing ATP production. This energy shortfall led to the downregulation of key markers essential for dopaminergic cell differentiation and maturation, indicating that an adequate purine salvage mechanism is vital for the proper development of dopaminergic neurons.

Kamatani et al. (2017) reported that the simultaneous oral administration of febuxostat and inosine elevated both hypoxanthine and ATP levels in the plasma. Interestingly, in two patients with mitochondrial disease, co-administration of febuxostat and inosine improved heart failure markers and insulin response, suggesting potential therapeutic benefits of increasing hypoxanthine in addressing mitochondrial impairment (Kamatani et al., 2019). Additionally, we demonstrated that elevating hypoxanthine levels through the co-administration of febuxostat and inosine ameliorated symptoms of patients with PD in a single-arm, open-label trial (Watanabe et al., 2020).

The loss of one hypoxanthine molecule leads to the loss of one IMP molecule. Consequently, if the PPP is utilized to synthesize IMP, which requires up to seven ATP molecules, the overall ATP balance may be affected negatively. Furthermore, the use of G6P in the PPP, instead of glycolysis, could further reduce ATP production. Therefore, optimizing hypoxanthine levels may help support dysfunctional mitochondria by introducing a metabolic shift away from the PPP.

**Figure 2:**
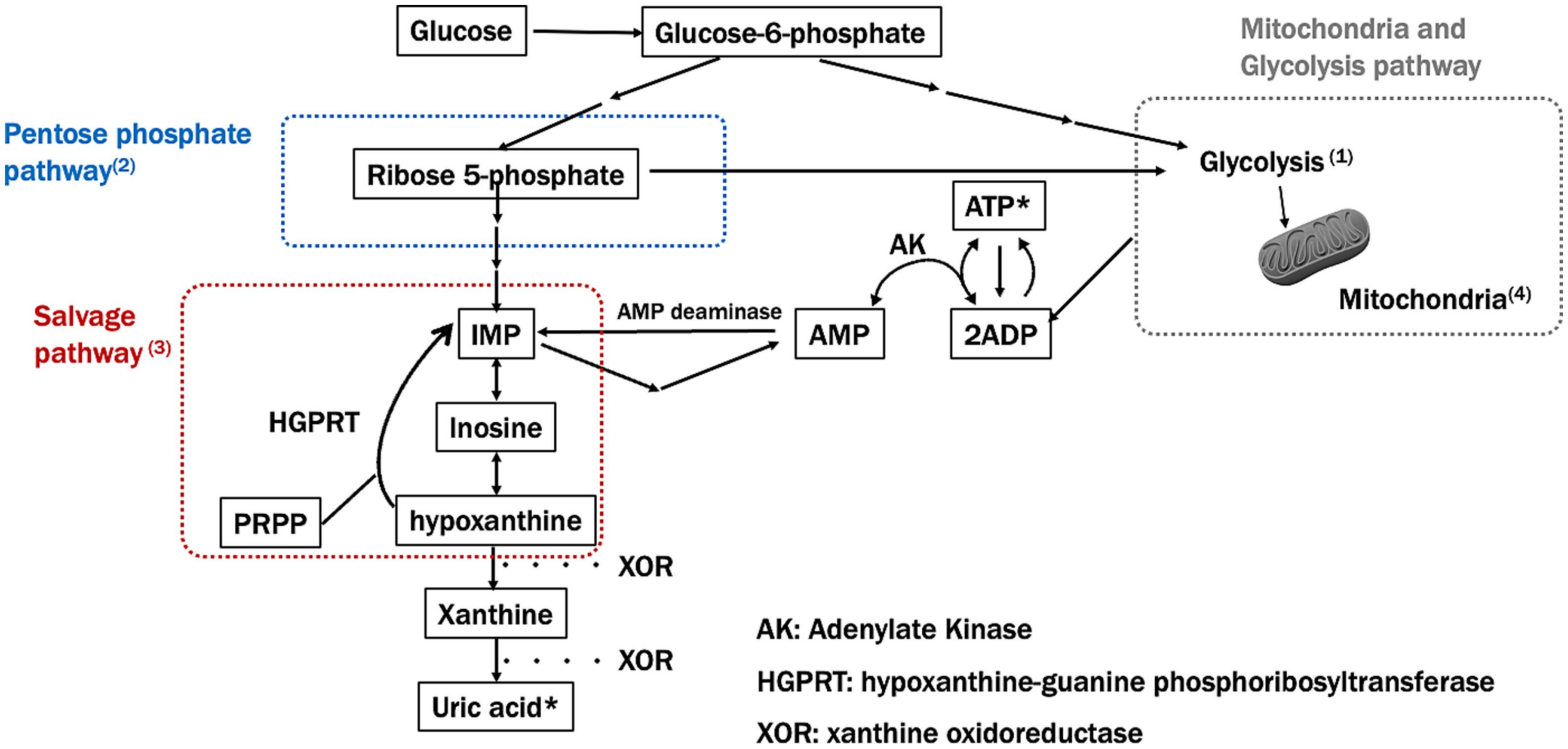
Major ATP synthesis pathways and impairment in Parkinson’s disease (PD). This figure shows the key pathways involved in ATP synthesis, specifically focusing on the pentose phosphate and salvage pathways and how these are impaired in Parkinson’s disease (PD). Numbers in the figure correspond to significant findings and their implications in PD pathology: (1) Glucose-6-phosphate (G6P) and Glycolysis: impaired glycolysis reduces ATP production in PD, as mitochondrial dysfunction prevents efficient energy production. (2) Ribose-5-phosphate and PRPP synthesis: Impairment in the pentose phosphate pathway (PPP) limits the production of precursors needed for nucleotide synthesis, further depleting ATP levels in PD. (3) Salvage pathway: hypoxanthine, a key intermediary in the salvage pathway, is essential for regenerating IMP and subsequently ATP. A reduction in hypoxanthine may impact both the PPP and mitochondrial function, potentially worsening ATP depletion in PD. (4) Mitochondrial ATP production: mitochondrial dysfunction, a hallmark of PD, disrupts efficient ATP synthesis through oxidative phosphorylation, leading to energy deficits.

In summary, major ATP synthesis pathways are widely impaired in PD (Figure 2). The PPP, glycolysis, and salvage pathways may interact with each other and play a broad role from the onset to the progression of PD. To deepen our understanding, it will be necessary to comprehensively analyze the relationships between these pathways, utilizing mathematical models and other approaches.

### Disruptions in ATP homeostasis and *α*SN aggregations

Hydrotropes are small molecules that solubilize hydrophobic substances in water and typically function at high concentrations. ATP is known to act as a biological hydrotrope, capable of both preventing the formation of and dissolving protein aggregates at physiological concentrations of 5–10 mm (Patel et al., 2017). This suggests that beyond its role as an energy source, ATP may help maintain protein solubility in cells, which could explain its high cellular concentrations. Takaine et al. (2022) showed that AMP-activated protein kinase (AMPK) and adenylate kinase (ADK) work together to maintain ATP levels, regardless of glucose availability. Disruptions in ATP homeostasis led to transient depletions, promoting *α*SN aggregation. Importantly, increasing ATP levels prevented these toxic aggregates, indicating that AMPK and ADK can contribute to preventing proteinopathies, such as PD.

Nuclear magnetic resonance spectroscopy, fluorescence, dynamic light scattering, and microscopy have revealed that ATP disrupts intramolecular contacts in *α*SN monomers, promoting initial aggregation and inhibiting the formation of late-stage fibrils (Kamski-Hennekam et al., 2023). ATP binds to specific regions of *α*SN, altering its conformational state and reducing its propensity for aggregation. These effects, influenced by magnesium ions and PD-related mutations, suggest that changes in ATP’s interaction with *α*SN may contribute to PD development.

ATP depletion also impairs autophagy by hindering the formation and elongation of autophagosomes and their fusion with lysosomes—processes that require substantial energy (Mandic et al., 2024). Additionally, failure of ATP-dependent proton pumps disrupts lysosomal acidification and reduces the activity of degradative enzymes. Consequently, the clearance of cellular debris and damaged organelles is compromised, leading to a loss of cellular homeostasis. Further studies are needed to elucidate the relationship between ATP depletion-related autophagy/proteasome dysfunction and *α*SN aggregation.

A study using an optogenetic tool to control *α*SN aggregation revealed that the induced aggregates transiently interacted with mitochondria, causing mitochondrial depolarization, reduced ATP production, and mitochondrial degradation through cardiolipin externalization-dependent mitophagy (Lurette et al., 2023).

Communication between the endoplasmic reticulum (ER) and mitochondria is critical for cellular homeostasis, including calcium signaling, lipid metabolism, and mitochondrial dynamics in neurodegenerative diseases. It was found that *α*SN binds to VAPB, an ER membrane protein, disrupting its interaction with PTPIP51, a mitochondrial membrane protein (Paillusson et al., 2017). The VAPB-PTPIP51 complex serves as a tether between the ER and mitochondria, facilitating essential processes, such as calcium exchange and ATP production. Disruption of these tethers by *α*SN, also seen in neurons from familial PD patients, impairs ER-mitochondria communication, leading to defective calcium signaling and reduced mitochondrial ATP production, both of which are crucial for neuronal health (Paillusson et al., 2017).

In summary, disruption of ATP homeostasis plays a multifaceted role in *α*-synuclein aggregation and the progression of Parkinson’s disease. ATP not only serves as a biological hydrotrope, preventing protein aggregation, but also supports essential cellular processes such as autophagy and ER-mitochondrial communication, both of which are crucial for neuronal health. Evidence suggests that restoring ATP levels and their interactions with *α*-synuclein could be key in preventing proteinopathies, although further research is necessary to fully understand the underlying mechanisms and the therapeutic implications.

### Proposed model of brain energy maintenance and its disruption in PD

According to Friston’s Free Energy Principle (Friston, 2010), the human brain is a highly energy-intensive organ that requires substantial amounts of ATP to maintain low entropy levels and support critical functions such as protein quality control and degradation.

In the healthy brain, ATP is continuously synthesized and utilized to ensure the clearance of pathological proteins, minimize free energy, and maintain cellular homeostasis. The proteolytic activity of ATP also plays a crucial role in maintaining protein quality control (Hipp et al., 2019), thereby reducing entropy. These energy-dependent mechanisms work in tandem to preserve the brain’s network efficiency and structural integrity, which are essential for complex functions. However, with aging and the onset of neurodegenerative diseases such as PD, there is a significant decline in ATP production, leading to a failure in entropy regulation and the progression of neurodegenerative pathology.

As already discussed, several factors contribute to this energy imbalance, including mitochondrial dysfunction, impaired glycolysis, neuroinflammation, and oxidative stress. In the context of PD, research has shown that disruptions in the brain’s energy supply primarily affect hub regions rich in synapses and long axons, which are critical for various higher brain functions. The brain’s ability to conduct adequate quality control is compromised as ATP levels decrease. This results in the accumulation of pathological proteins, such as misfolded *α*-SN, further elevating the brain’s entropy levels and perpetuating a cycle of energy crisis and protein aggregation.

Furthermore, the decline in ATP levels impacts the brain’s capacity to manage cellular processes such as autophagy, which is crucial for removing damaged organelles and protein aggregates. Under healthy conditions, a large amount of ATP is allocated for the degradation of such waste products, preventing the onset of neurodegenerative pathways. However, reduced ATP availability can impair these processes in a diseased state, leading to toxic aggregates and neuronal dysfunction (Mandic et al., 2024).

The decline in ATP is not solely due to mitochondrial impairment but also involves disruptions in other metabolic pathways, such as the PPP and ATP recycling mechanisms, as discussed in this review. The decline in ATP disrupts the brain’s quality control mechanisms, leading to impaired autophagy and the inability to degrade pathological proteins, such as *α*SN. This accumulation of *α*SN not only drives the progression of PD but also exacerbates other pathological processes, such as oxidative stress, neuroinflammation, and the breakdown of neuronal network integrity.

**Figure 3:**
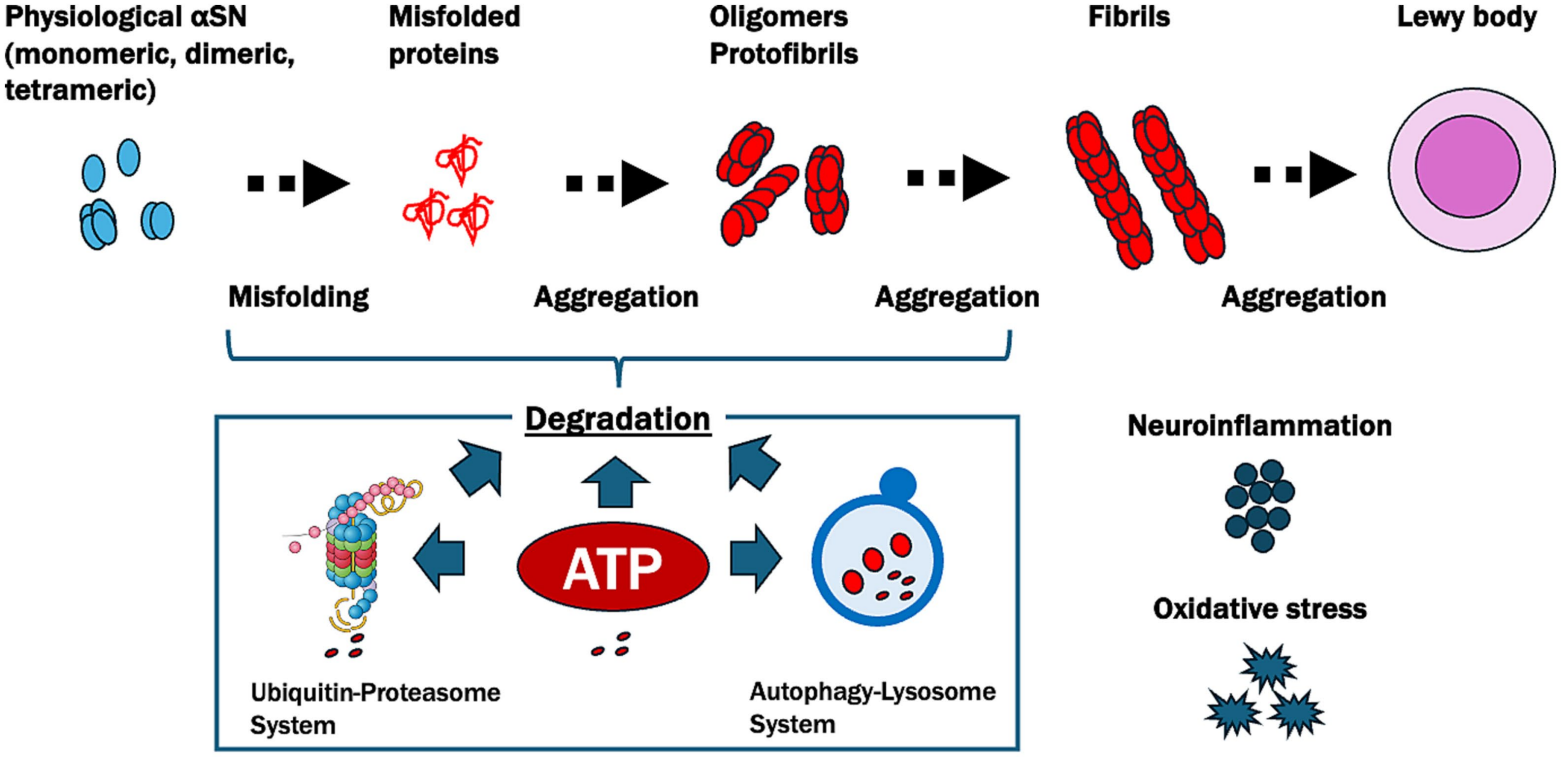
Pathways of *α*-synuclein aggregation and ATP-related degradation in Parkinson’s disease (PD). This figure illustrates the progressive misfolding and aggregation of *α*-synuclein (*α*SN), a protein central to PD pathology, and highlights the crucial role of ATP in its degradation. Physiological *α*SN usually exists in monomeric, dimeric, or tetrameric forms. However, under pathological conditions, it undergoes misfolding and aggregates into oligomers, protofibrils, and fibrils, eventually forming Lewy bodies. ATP plays a pivotal role in degrading these misfolded and aggregated *α*SN proteins through two main pathways: the ubiquitin-proteasome system, which relies on ATP for the tagging and degradation of smaller misfolded proteins, and the autophagy-lysosome system, which is responsible for breaking down larger aggregates like fibrils and requires ATP to facilitate the formation, transport, and fusion of autophagosomes with lysosomes. In PD, reduced ATP levels due to oxidative stress and neuroinflammation impair these degradation pathways, leading to the accumulation of toxic aggregates and contributing to neurodegeneration.

Based on these findings, we proposed the ATP supply–demand mismatch model in the pathogenesis of PD (Figure 3). This model emphasizes the central role of ATP homeostasis in PD, suggesting that therapeutic strategies to restore ATP levels could help mitigate *α*SN aggregation and its downstream effects, potentially providing a targeted approach to slowing or halting disease progression.

**Figure 4:**
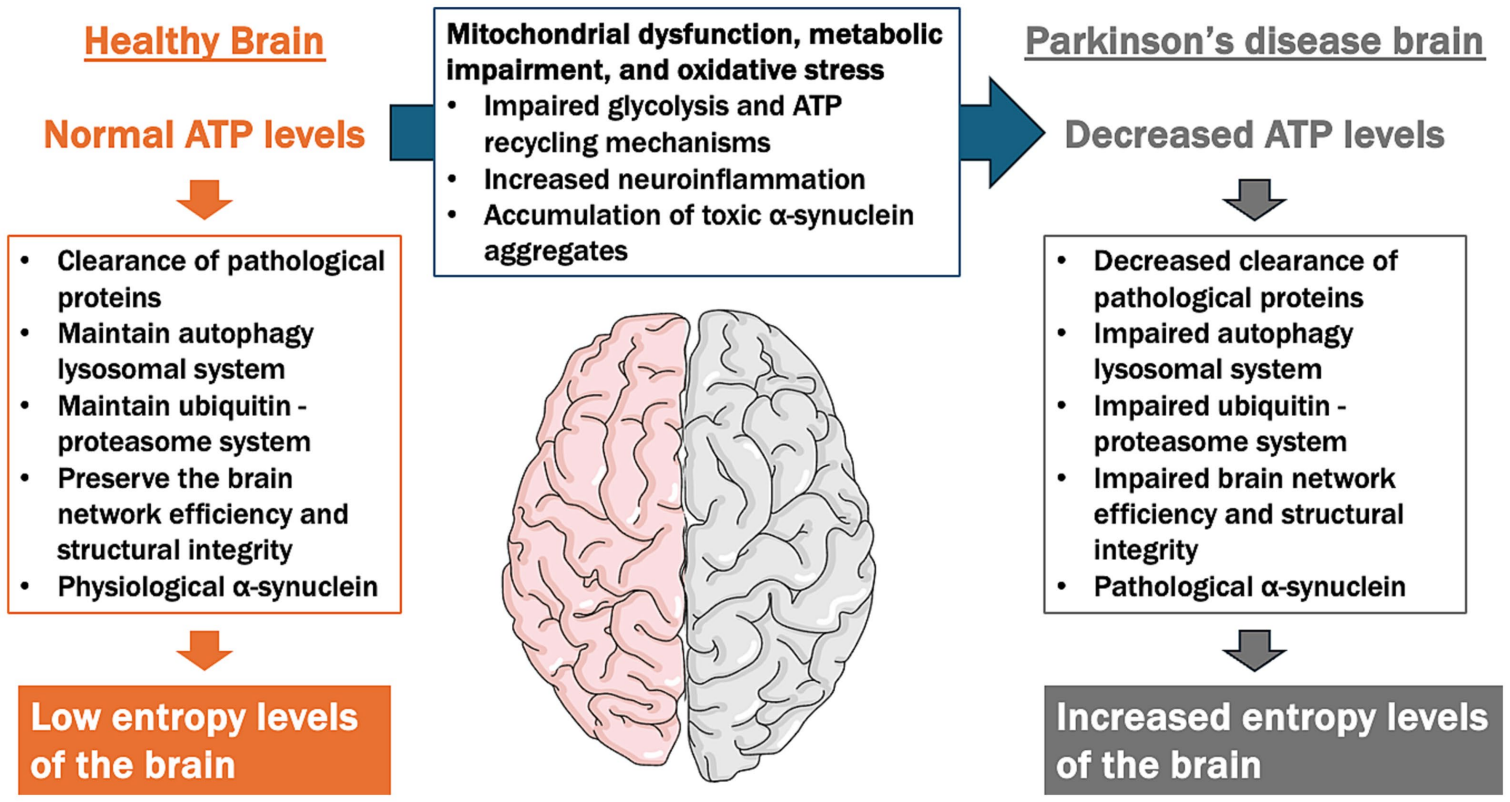
ATP supply–demand mismatch model in the pathogenesis of Parkinson’s disease (PD). This figure illustrates the contrast between ATP levels and cellular functions in a healthy brain versus a PD brain, focusing on the imbalance between ATP supply and demand contributing to neurodegeneration. In a healthy brain, normal ATP levels support essential cellular functions, including the clearance of pathological proteins, the maintenance of the autophagy-lysosomal and ubiquitin-proteasome systems, and the preservation of brain network efficiency and structural integrity. This results in low entropy levels, indicating efficient cellular homeostasis. In contrast, the PD brain is characterized by decreased ATP levels due to mitochondrial dysfunction, impaired glycolysis, reduced ATP recycling mechanisms, and increased oxidative stress and neuroinflammation. These factors lead to impaired clearance of pathological proteins, dysfunction in autophagy-lysosomal and ubiquitin-proteasome systems, and disrupted brain network integrity. As a result, high entropy levels emerge, reflecting a breakdown in cellular organization and function, which contributes to the progression of neurodegeneration in PD.

Importantly, the ATP Supply–Demand Mismatch Model may also extend to other neurodegenerative diseases characterized by energy deficits and abnormal protein accumulation, collectively known as proteinopathies. For instance, Alzheimer’s disease is associated with mitochondrial dysfunction, disrupted glucose metabolism, and the accumulation of amyloid-beta and tau proteins, which could both result from and exacerbate ATP deficits (Watanabe et al., 2021). Similarly, amyotrophic lateral sclerosis involves impaired energy metabolism and mitochondrial dysfunction, further contributing to neuronal vulnerability. Given the pivotal role of ATP homeostasis in regulating protein quality control, autophagy, and neuronal network integrity, this model may provide a unifying framework for understanding common pathological mechanisms across neurodegenerative diseases. Future research exploring shared pathways in these conditions could help refine and validate the broader applicability of this hypothesis (Figure 4).

## Conclusion

In PD, the clinical features are better understood at the network level rather than as localized lesions. Additionally, several lines of evidence indicate that energy deficits play a role in both motor and non-motor symptoms. This review highlights the central role of ATP reduction and hypoxanthine deficiency in the pathogenesis of PD. Impaired protein quality control and autophagy lead to *α*-synuclein accumulation and neurodegeneration. Therapeutic strategies to restore ATP production and optimize hypoxanthine levels may mitigate these pathological processes, offering promising avenues for slowing disease progression.
